# Risk Assessment of High-Speed Rail Projects: A Risk Coupling Model Based on System Dynamics

**DOI:** 10.3390/ijerph17155307

**Published:** 2020-07-23

**Authors:** Yutong Xue, Pengcheng Xiang, Fuyuan Jia, Zhaowen Liu

**Affiliations:** 1 School of Management Science & Real Estate, Chongqing University, Chongqing 400045, China; xueyutong_cn@163.com (Y.X.); 20180301006@cqu.edu.cn (F.J.); 2 International Research Center for Sustainable Built Environment, Chongqing University, Chongqing 400045, China; 3 Construction Economics and Management Research Center, Chongqing University, Chongqing 400045, China; 4 Faculty of Civil Engineering and Geosciences, Delft University of Technology, 2628CN Delft, The Netherlands; z.liu-8@tudelft.nl

**Keywords:** high-speed rail, risk assessment, dynamic risk, risk coupling, risk interaction, system dynamics

## Abstract

Due to their characteristics and multiple objectives, high-speed rail (HSR) projects carry more complex risks than conventional projects and high correlation and conductivity are among the associated risk factors. Previous risk assessment frameworks for rail infrastructure have ignored the effects of risk interactions that inflate risk levels, namely, risk coupling effects. Based on a system dynamics method, this paper develops a risk coupling model for HSR project risk assessments. A risk factor list is established from a literature review, and relationships analysed using a case study and expert interviews. System dynamics equations are constructed and their parameters obtained by expert evaluations of risk factors. The proposed model is applied to a real-world HSR project to demonstrate it in detail. The model can evaluate the risk levels of HSR projects during a simulation period. In particular, it can identify the key coupling effects that are the main increased risk. It provides a significant resource, using which HSR project managers can identify and mitigate risks.

## 1. Introduction

In recent years, China’s rapid development has benefited from large-scale infrastructure construction projects, one of which is the high-speed rail (HSR) project [1]. Since 2008, the scale of the HSR network that China has put into operation has exceeded the sum of all other HSR networks in the world. The Mid-to-Long-Term Railway Network Plan (revised in 2016) proposes further improvements to the HSR network: by 2030, there should be eight vertical and eight horizontal corridors (called the ‘‘8+8” network) with expanded regional railway connections constituting a total length of 45,000 km. While the high construction demands of HSR projects create development opportunities, they also bring great challenges to project managers.

Chinese HSR projects have unique characteristics. On the one hand, they require complicated infrastructure such as tracks, tunnels and bridges which, due to their high quality standards, create difficult technical problems. For example, the project managers of the Beijing-Shanghai HSR needed to employ construction technology such as high-speed, long-span, deep-water bridges, ballast-less tracks, the laying and welding of long tracks, and track vibration mitigation. In order to solve these problems, HSR projects require huge investments that are two–three times those required for normal train systems [2]. Moreover, the organizational management of HSR projects is difficult because the projects span several administrative regions and include stakeholders at multiple levels, such as central and local governments, project developers, experts and contractors. On the other hand, HSR projects, as a typical type of major infrastructure project, bring huge externalities to the environment, economy and society. Compared with general engineering projects, HSR projects have multiple objectives [3,4]: (1) First, within the project system, the traditional project management objectives of investment, quality, duration and safety should be achieved [5]. (2) Secondly, the HSR project is intended to promote regional economic development along the train line and avoid ecological damage, health hazards and social injustice. Therefore, the external objective of the HSR project is to achieve sustainable performance in the economy–ecology–social system [6].

Risk can be measured by the probability and consequence of not achieving a defined project objective [7,8] and its occurrence is often accompanied by certain losses [9]. Obviously, the characteristics and multiple objectives of HSR projects make their risks more complicated [10]. Once a loss occurs, project benefits may be lost, triggering regional economic disparity, ecological destruction, and even social conflict [11]. Therefore, it is of high importance to carry out risk assessment for HSR projects. Previous research on risk assessment in major infrastructural or rail megaprojects has been conducted. Chapman presented a theoretical framework for examining the dimensions of complexity in rail projects. According to his study, complexity lies in the six dimensions of finance, context, management, site, task and delivery [12]. Dong proposed an integrated risk evaluation model to evaluate the economic, social and ecological risks of a certain HSR project [13]. Wang put forward an Analytic Hierarchy Process (AHP)-based major infrastructure risk assessment framework, where environmental risks, project implementation risks and decision-making-behaviour risks are taken into account [14]. Despite their contributions, these risk assessment tools are mostly static and the risks are considered independently. However, the HSR project is a complex system with multiple interrelated subsystems. There are dynamic relationships between project and environmental components that are mainly reflected in their mutual constraints and support [6]. Furthermore, the components of the project are subject to change as part of a dynamic coordination process [15]. Such dynamic complexity makes the risks of HSR projects highly interrelated, and the risks are conductive within and between the internal and external environments of the system [16]. Ignorance of the potential interactions between risks may mean that project managers may not fully understand these risks, thus reducing the efficiency and quality of risk management [17]. Furthermore, because interactions may change the intensity and type of risk, the total risk of the HSR system cannot be calculated by a simple linear superposition of the probabilities of risk factors. Therefore, it is necessary to develop a dynamic risk assessment model for risk assessment in HSR projects, especially one that considers the effects of risk interactions.

In physics, coupling refers to the phenomenon in which two or more systems or factors influence each other by interacting. In the energy and electricity field, the essence of coupling is defined as the transfer of energy from one entity to another through such interaction [18]. In this paper, we introduce the concept of coupling into the field of project risk management and focus on risk coupling effects in HSR projects. A risk coupling effect is defined as an increase in risk due to risk interactions. To be specific, over its lifecycle, the HSR project will inevitably be disturbed and affected by uncertain factors within the internal and external environment of the system, resulting in a deviation between the actual situation and the target of a project node; that is, risk occurs. Then, risk spreads and transmits throughout the project system through paths including stakeholder relationships, business processes and the project lifecycle. When there are multiple risks transmitted through the project, their interactions may lead to rapid increases in risk or the creation of new risks, thus increasing negative impacts that could cause the HSR project to fail to achieve its objectives and/or suffer irreparable losses. In order to account for coupling risk in HSR project risk assessments, a system dynamics model is developed to describe and simulate risk coupling effects qualitatively and quantitatively. The goal of the model is to evaluate risk levels and identify the key risk coupling effects in HSR projects. The evaluation model can be used for pre-, stage- and post-evaluations of certain HSR projects. The findings provide an important decision-making reference for managers of HSR and other major infrastructure projects.

## 2. Literature Review

### 2.1. The Risks for the High-Speed Rail Project

The first step in assessing the risks of the HSR project is to identify the risk factors. Previous risk evaluation frameworks of major infrastructure projects provide some reference. However, due to the characteristics of HSR projects, the indicators in these frameworks are not fully applicable. Combined with studies on risk in specific HSR project fields, we integrate the risks related to HSR projects and classify them into two risk subsystems. According to Haimes, the occurrence of risk comes from the combination of internal vulnerabilities and external threats to the system [19]. That is, due to the vulnerability of the system, the probabilities and effects of risks will be triggered by man-made or natural threats. Risk assessment methods based on external threats and internal vulnerabilities have been widely used [20,21]. Following this method, this paper divides HSR project risks into two risk subsystems: internal vulnerabilities and external threats, and elaborates on their types and their risk factors. Our study of the HSR project’s risks considers factors that affect its objectives during its life-cycle, including the decision-making, tendering and bidding, design, construction and operating stages. Facility risks and safety risks such as leakage, fire and explosion during the operating phase are beyond the scope of this paper.

#### 2.1.1. Vulnerability Risk

Vulnerability risk refers to risk to the internal environment of the project. The internal environment contains elements related to the characteristics of HSR projects (large investment scale, technical difficulty and multiple participants) and the activities of core stakeholders (such as central and local governments, developers and contractors). Classified according to factors of production, the vulnerability risk of HSR projects exists in three fields: capital, technology and management.

Among the capital risks, in developing countries such as China the government, as the main HSR investor, experiences a large financing gap concerning railway construction. Moreover, it is difficult to carry out market-oriented financing during its life-cycle, especially in the investment decision stage [22,23]. Some HSR projects put high demands on the government’s capital supply and, due to the conflict between increasing public investment and limited revenue, debt financing has fueled a boom in government debt [24]. When the HSR debt of local governments reaches a certain scale, construction funds may not be able to be provided on time. In addition, because HSR projects have long life-cycles and are owned by the public sector, they are often accompanied by a certain degree of corruption in the decision-making and tendering and bidding stages [25]. Compared with Japan and other countries, the relatively low fares on China’s HSR make it difficult for long-term operational cash flow to balance the interest incurred by high investment during the operating phase [26]. These factors constitute a capital risk to HSR projects.

In terms of technology, the variety of complex structures required for HSR projects need complex construction techniques and high levels of construction safety [27,28,29,30]. In addition, in the preliminary design stage, there may be situations where the accuracy of surveys of geology, landforms and hydrology is insufficient, or engineering designs are flawed [31]. Since HSR projects often span several administrative regions, differences in technical standards within the same project are likely in the design stage [7]. These factors contribute to the technical risk of HSR projects.

Management adds another dimension to vulnerability risk. In Chinese HSR projects, there may be defects in the decision-making system, such as no scientific basis for assessing the priorities of new HSR corridors [32,33] or a lack of public participation in the decision-making process [34]. On the other hand, there is an oligopoly in railway design, construction and consulting services. Lack of competition and external supervision increases the moral risk of contractors, which may lead to illegal subcontracting, “Jerry-building” and fraud [35]. In addition, because a project may span several regions, the participants (especially local governments) are prone to poor communication and conflicts of interest during its life-cycle [36]. Insufficient professional abilities, especially among construction workers with high job mobility, also contribute to management risk [37,38]. To sum up, the risk factors above constitute the vulnerability risk within the internal project environment. They make it difficult for HSR projects to achieve the internal objectives of cost, quality, schedule and safety.

#### 2.1.2. Threatening Risk

Threatening risk represents the risk within the project’s external environment [39]. Relative to vulnerability risks, they are more difficult to predict and mitigate. Corresponding to the multiple objectives of HSR projects, the external threats lie in three aspects: ecology, economy and society.

To allow faster travel than on conventional railways, HSR railways require fewer bends and gentler slopes, which pose greater threats to the ecological environment along the route during construction and operation. Especially in areas with highly vulnerable ecosystems, solid waste, wastewater, noise and vibration generated by HSR projects are more likely to damage the ecosystem [40]. Natural disasters such as typhoons, heavy rains, earthquakes and floods also pose threats to ecological security [41]. Complex hydrological, geological and meteorological conditions [42,43], as well as high environmental requirements for nature reserves and historic reservations along the route in the decision-making, design and construction stages [44], may make it difficult to achieve the ecological sustainability goals of HSR projects.

From the perspective of economy, unfavorable economic situations will cause adverse changes in the market [13]. In economically-underdeveloped regions with low per-capita GDP, small populations and low regional attractiveness have negative impacts on HSR usage [45]. Additionally, there is evidence that the “siphon effect” brought by HSR tends to benefit large-scale transport hub cities rather than small cities and counties [46,47]. This means that the HSR may cause excessive concentrations of capital elements according to levels of economic development along the route. Besides, alternative transportation facilities, such as air and road, can reduce the market share of HSR projects [48]. Such factors are not conducive to HSR projects meeting expected market demand and promoting regional economic growth during operation.

In terms of social risks, wars and riots are considered social risk factors [14,49]. In particular, land acquisition, demolition and resettlement work related to major infrastructure projects may cause social conflicts in China [50,51,52]. In addition, some residents and communities are sensitive to the pollution caused by HSR, which will lead to NIMBY (not in my backyard) conflict in the decision-making stage [53]. Besides, when the public expresses their strong positive opinions towards HSR projects, this can cause social conflict [33]. Table 1 summarizes the risks to HSR projects based on the reviewed literature. Note that some of these risks are primarily based on HSR projects in China, as described above.

### 2.2. Risk Assessment Methods and System Dynamics

After risk identification, a risk assessment method should be selected. An appropriate method helps improve the accuracy of the assessment and the understanding of HSR project risks. Methods used for risk assessment in construction projects include the analytic hierarchy process, fuzzy comprehensive evaluation, Delphi, Monte Carlo simulation, system dynamics, etc. [14,17,54,55,56,57,58,59,60,61]. Table 2 shows some general methods commonly used for risk assessment in construction projects.

As mentioned above, in this paper we need to choose a method that can consider the dynamic relationships between the project’s risk factors [16,62]. Of the methods summarized in Table 2, the Bayesian network, interpretative structural modeling, N-K model, and system dynamics (SD) methods meet this requirement. Among these, interpretative structural models focus more on qualitative results to provide a theoretical framework. Meanwhile, the SD, Bayesian network and N-K model methods can be used to study risk coupling in both a qualitative and a quantitative manner, and so are more applicable to the present study. However, the N-K model and Bayesian network methods require large amounts of sampled data, making them unsuitable for the present study of risk couplings in HSR projects. Moreover, SD models can simulate the risk status of a project during a period of time, which satisfies the predictive requirement of risk assessment. Therefore, we selected the SD method for modelling the risk couplings in HSR projects.

The SD method was proposed by Professor J. W. Forrester in the United States. In recent years, it has become a pivotal approach to modeling the interrelationships and feedbacks that exist within complex systems [63]. It has reached maturity in applications to risk management in construction projects. For example, based on SD, Wang developed a systems-based safety risk model that takes into account the interaction between organizational processes and technical systems, and demonstrated it on an urban metro tunnel project [64]. Through SD, Nasirzadeh modeled the interdependent components and external interactions of machine breakdown risk and quantified the full impact of this risk on the duration and cost of a bridge construction project [65]. Generally speaking, the steps of system dynamics modelling include [59,60]:

1. System Analysis. To articulate the problem and determine the boundary of the system and subsystem; that is, to determine the scope of the study.
2. Causality diagram and flow-stock diagram. To analyze the relationships between the system components and qualitatively describe these in a causality diagram. On this basis, the components are defined as different variable types, which are displayed in the flow-stock diagram.
3. Establishing SD equations. This step designs functions to clarify the quantitative relationships among variables in the system and estimate parameter values for use in the equations.
4. Simulation and evaluation. After setting the simulation time and step size in the SD software, the status of each variable and the whole system can be simulated within a period of time or at a certain time node. Further, the simulation results can be compared by varying the values of the variables in the SD equations.

## 3. Methodology

**Figure 1:**
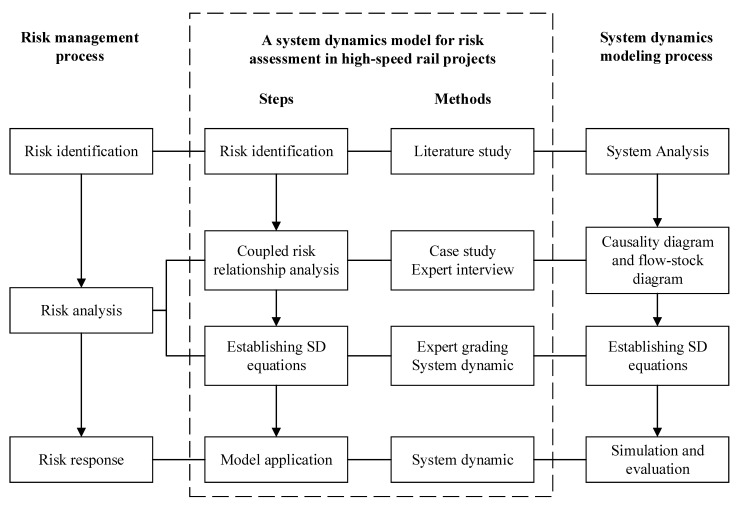
Research framework.

Combining the steps of risk management with the SD modelling process, this paper will follow the logical framework of the four phases shown in Figure 1.

First, we identify risks related to HSR projects by a literature review. Based on the classification of vulnerability and threatening risk subsystems, 24 risk factors and six risk fields were identified.

Second, the coupled risk relationships are preliminarily constructed based on case studies. Then, these relationships are revised and supplemented based on the results of one-on-one interviews with three university scholars engaged in HSR research. Thus, a comprehensive causality diagram and flow-stock diagram are established.

Third, the mathematical relationships between system variables are clarified based on SD theory, and the parameters involved are determined by the expert grading method. For this, questionnaires were sent to senior managers participating in the project. Thus, SD equations are established.

Finally, the model is applied to a typical HSR project, the Zhengzhou-Wanzhou (ZW) HSR, to simulate over a certain period of time. The risk level and key coupling risk effects are determined, and a risk control strategy is proposed.

## 4. Coupled Risk Relationship Analysis

**Figure 2:**
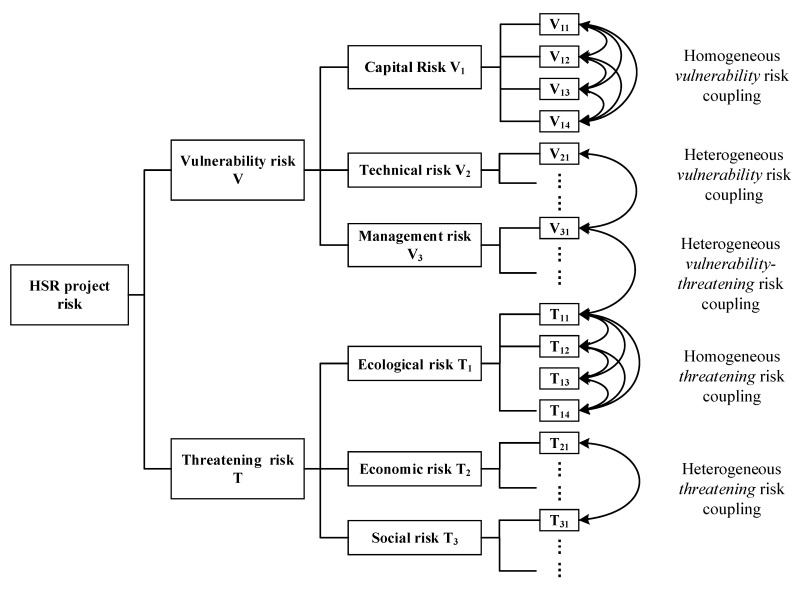
Schematic diagram of coupled risk relationships in high-speed rail (HSR) projects.

Based on the risk list in Table 1, and according to cases and expert interviews, this section qualitatively analyses the relationships between the coupled risks, which are essentially the causal relationships between risk factors. First, we analyze the internal coupled risk relationships in the vulnerability and threatening subsystems, then discuss the coupled risk relationships between the two subsystems. For the convenience of expression, coupling between risk factors within a certain risk category (such as the coupling of capital risk factors) is called homogeneous risk coupling, while coupling between risk factors of different categories (such as coupled capital and technical risk factors) is called heterogeneous risk coupling. The different types of risk coupling are shown in Figure 2.

### 4.1. Risk Coupling in the Vulnerability Risk Subsystem

#### 4.1.1. Homogeneous Coupling of Vulnerability Risks

First of all, we analyze the coupled relationships of homogeneous risk factors within a single risk category (technology, capital and management).

Coupling of technical risk factors. Survey and design defects can mean that the planned construction technology fails to meet the project’s needs, further increasing the complexity of the required construction technology. These defects, or technological complexity, may lead to inadequate safety protection measures during the construction process, resulting in accidents. For example, in the Guiyang-Nanning HSR project, due to insufficient assessment of the geological conditions that determine the stability of its rock walls, inadequate support measures were taken, resulting in a large area of collapse.

Coupling of capital risk factors. In China, the relatively low fares set for HSR have led to insufficient operating profitability. Except for a few lines, such as the Beijing-Shanghai and Beijing-Tianjin lines, most HSR lines operate at a continuous financial loss. Therefore, as the main investor and financial supporter, the government has increased its debt risk. Besides, audit cases show that senior managers have committed corruption and bribery, such as embezzlement of public funds and setting illegal charges. For example, in the funding process of the Beijing-Shang HSR, 11 senior government leaders misappropriated public funds and construction funds totaling 187 million yuan. This aggravated breaks in the project capital chain, thus increasing the difficulty in financing.

Coupling of management risk factors. Insufficient ability and experience of project managers may cause inadequate demonstration of project schemes and irrational decisions. Additionally, once there are many participants with conflicting interests, the moral hazard of corporate defaults is likely to occur.

#### 4.1.2. Heterogeneous Coupling of Vulnerability Risks

The heterogeneous coupling relationships between the three types of vulnerability risk are more complicated. The occurrence of any kind of risk may induce the successive occurrence of other risks. Combined with the homogenous risk coupling described in Section 4.1.1, risk coupling paths are formed within the vulnerability risk subsystem. The main risk coupling paths (causality chains) are briefly described below.

Causal chain 1: In the China-Russia-Mongolia HSR project, the standard track gauge used in Mongolia and Russia is different from that of China. To solve this problem, China has developed a track system with two sets of rails that can be used in all three countries. Complex technology has led to an increase in human resources and mechanical equipment. The cost of engineering construction investment has overrun, increasing financing difficulties. With limited funds, project managers and construction personnel with sufficient professional experience cannot be hired, thus causing safety problems. This chain can be summarized as: Different technical standard → Technical complexity → Financing difficulty → Insufficient member ability → Inadequate safety protection.

Causal chain 2: At present, in China, decision-making related to HSR stations and lines is led by experts from the Ministry of Railways of the central government; however, investment in the construction phase and the regional socioeconomic benefits of the operational phase are closely related to local governments. For the sake of maximizing their own interests, there is often interest gaming among local governments during HSR planning phases. The rent-seeking behaviour of local government officials is likely to lead to bribery and corruption in decision-making departments. In turn, the decisions made by the decision-making department may be affected by interest bias. This can be summarized as: Decision defect → Conflicts of interest → Corruption and bribery → Decision defect.

Causal chain 3: In the HSR decision-making system, the concentration of public power, insufficient market competition mechanisms, and poor regulation lead to corruption and bribery by government officials. Some marketers have established personal relationships with senior government officials to become project contractors. Many of these lack relevant experience and capabilities, leading to quality problems later in the project. This chain can be summarized as: Decision defect → Corruption and bribery → Insufficient member ability.

**Figure 3:**
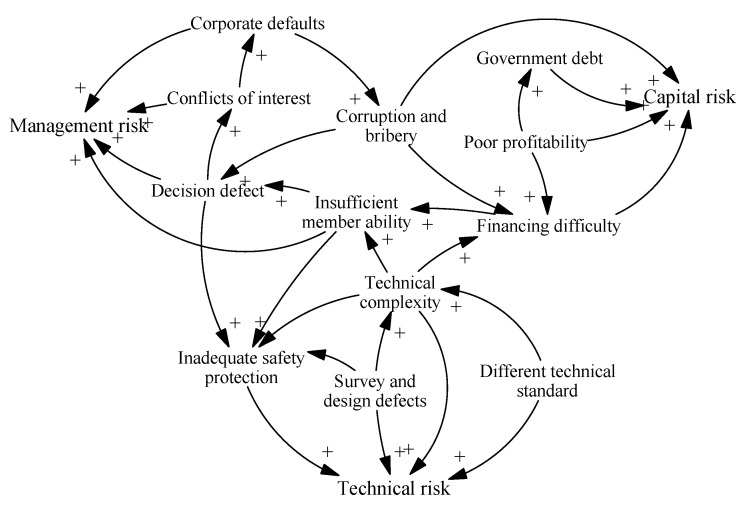
Risk coupling in the vulnerability risk subsystem.

Based on the analysis above, the risk coupling within the vulnerability risk subsystem of HSR projects is as shown in Figure 3.

### 4.2. Risk Coupling in the Threatening Risk Subsystem

#### 4.2.1. Homogeneous Coupling of Threatening Risks

Coupling of ecological risk factors. For areas with complex hydrogeological conditions, the excavation of HSR tunnels may damage the water and soil systems, making them more vulnerable to natural disasters such as landslides and debris flows [66]. Natural disasters can affect the diversity of animals and plants, thus destroying the balance and enhancing the vulnerability of the ecosystem.

Coupling of economic risk factors. Low levels of regional economic development have resulted in decreased demand for business and recreational travel and insufficient affordability. Although China’s HSR fares are lower than those of similar rail transit projects abroad, compared with ordinary train or road transportation HSR is aimed at medium- and high-earning customers. In this case, other transportation facilities may be chosen by people needing to travel, making the market share of HSR lower than that of the alternatives.

Coupling of social risk factors. During wars and riots, low social security is not conducive to the successful land acquisition, house demolition and resettlement works required for HSR projects. For example, due to armed conflict in the northern Myanmar region where the Kunming-Rangoon HSR is located, there were great difficulties in conducting house demolitions.

#### 4.2.2. Heterogeneous Coupling of Threatening Risks

**Figure 4:**
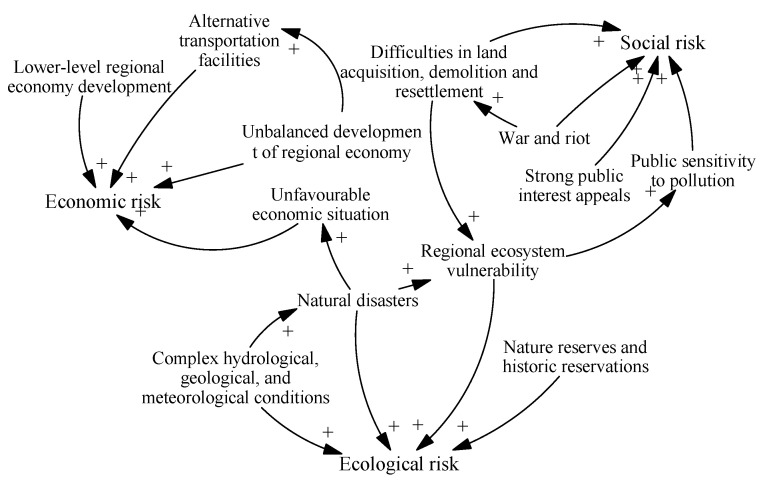
Risk coupling in the threatening risk subsystem.

The heterogeneous couplings in the threatening risk subsystem are relatively simple. They include the following causal relationships. First, in some cases, the vulnerability of the ecosystem along the route has increased the public’s risk perception of HSR projects. Second, the natural environment is greatly changed by large-scale land acquisition and demolition, increasing the vulnerability of the ecosystem. Third, serious natural disasters sometimes affect the macroeconomic situation by affecting the supply-demand structure. Fourth, wars and riots can affect the economic situation. For example, large-scale demonstrations held by Venezuela’s opposition parties aggravated an economic recession and interrupted the construction of HSR projects. The risk relationships within the threatening risk subsystem are summarized in Figure 4.

### 4.3. Risk Coupling Between the Vulnerability and Threatening Risk Subsystems

The heterogeneous coupling of the vulnerability and threatening risk subsystems is reflected in the availability of vulnerability risk factors to threatening risk factors, and the reaction of vulnerability risk factors to threatening risk factors. Based on the availability of data, we selected several cases, such as the Beijing-Shanghai HSR, to analyze the coupling between risk subsystems (Table 3).

**Figure 5:**
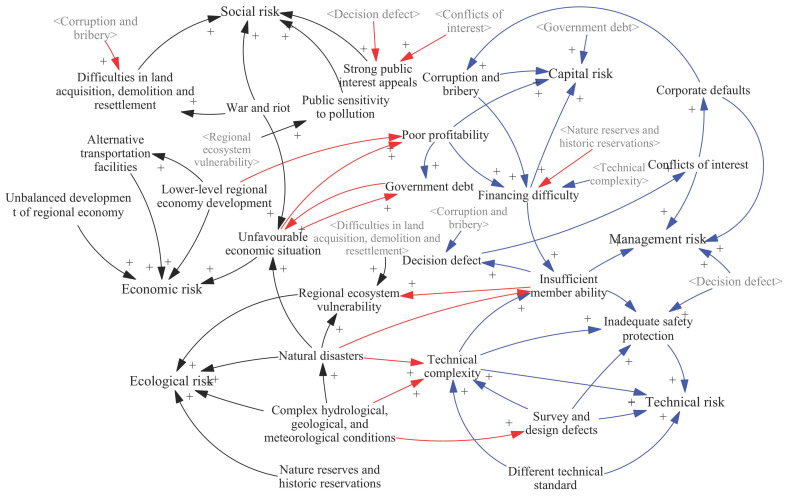
Causality diagram of risk couplings in the high-speed rail (HSR) project. Black lines represent couplings in the vulnerability risk subsystem, blue lines represent them in the threatening risk subsystem, and red lines represent the couplings between risk subsystems. The gray variables are used as the shadow variables of their corresponding black variables to keep the structure concise. They have the same function as their corresponding black variables.

Integrating the risk relationships described in Section 4.1, Section 4.2 and Section 4.3, a causality diagram of HSR project risks was obtained (Figure 5).

**Figure 6:**
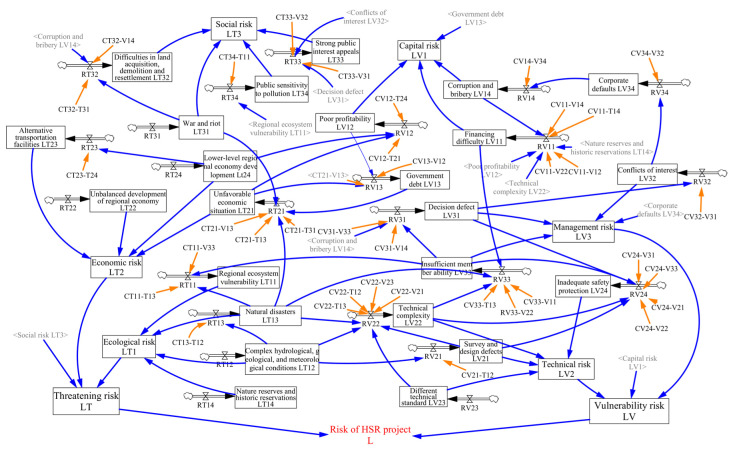
Flow-stock diagram of risk coupling in high-speed rail (HSR) projects. The gray variables are used as the shadow variables of their corresponding black variables to keep the structure concise. They have the same function as their corresponding black variables.

Figure 5 presents the structure of risk coupling in the project system. However, it cannot quantify the various risk factors and their coupling effects, so the risk level cannot be assessed. In fact, the coupling effects of risks are not only the process of risk evolution, but also the process of accumulating state variables (also known as stock). Therefore, our paper used Vensim PLE software to draw a system dynamics flow-stock diagram (Figure 6) on the basis of Figure 5. According to their nature, the factors in Figure 5 are distinguished as different variable types in Figure 6. For the risk level of various factors, if we take V_11_ as an example, LV_11_ is set as the state variable (stock) and its increase per unit time, RV_11_, is set as the rate variable (flow). At the same time, representing the risk level of a certain category, subsystem or whole HSR project, the levels of capital risk (LV_1_), technical risk (LV_2_), management risk (LV_3_), ecological risk (LT_1_), economic risk (LT_2_), social risk (LT_3_), threatening risk (LT), vulnerability risk (LV), and total risk (L) are set as auxiliary variables. In addition, since the coupling coefficients between risk factors will not change rapidly in a specific environment, they are set as constants.

## 5. Establishing System Dynamics Equations

In this section, SD equations are established according to the couplings between the risks and types of variables proposed previously. The system parameters are defined, including the weight of each risk factor, risk value and coupling coefficient.

### 5.1. Risk Assessment System and Weights of Indicators

The HSR project risk assessment system is established based on the risk list. The system is divided into four layers with corresponding indicators, including a target layer of the total risk of the HSR project, a standard layer formed by risk subsystems, a field layer formed by risk categories, and an index layer formed by risk factors. Indicators in each layer belong to the upper layer and are affected by indicators in lower layers.

To complete the risk assessment system, the indicator weights, including their absolute weights (indicating their importance to the target-layer indicator) and their relative weights (indicating their importance to the corresponding upper-layer indicator), should be determined. In this paper, the expert grading method was employed to determine them. Questionnaires were distributed to eight experts engaged in large-scale project management and risk management research at universities, who provided evaluations of the importance of each indicator at the index layer. Responses were made on a five-level Likert scale (1–5), where 5 represents high importance.

To reduce the subjectivity of expert experience to some extent, we processed the expert evaluations by averaging them. The mean score MS refers to the importance of $R_{ij}$ in the index layer. This, and its absolute weight $W_{R_{ij}}^{*}$, were calculated as follows:

(1) $$MS=\frac{\sum^{\text{​}}\left(f\;\times \;s\right)}{N}\;(1\leq MS\leq 5)$$

(2) $$W_{R_{ij}}^{*}=\frac{{MS}_{R_{ij}}}{\sum^{\text{​}}MS}\;(0\leq W_{R_{ij}}^{*}\leq 1)$$

where s is the expert rating (ranging from 1 = least important to 5 = most important), f is the frequency of rating (1–5) for each indicator (f∈ [0,8]), N is the total number of valid questionnaires (N=8), and $R_{ij}$ is the *j*^th^ index-layer indicator in the *i*^th^ field layer, where *R* ∈ {*V*,*T*}, (*i* = 1, 2, 3, 4), (*j* = 1, 2, 3, 4).

Then, the absolute weights of the indicators are normalized to obtain their relative weights. Specifically, the absolute weights $W_{R_{i}}^{*}$ of the field layer indicators are derived from the absolute weights $W_{R_{ij}}^{*}$ of the index layer indicators from Equation (3), and the relative weights $W_{R_{ij}}$ are derived from Equation (4).

(3) $$W_{R_{i}}^{*}=\sum_{j=1}^{n}W_{R_{ij}}^{*}$$

(4) $$W_{R_{ij}}=\frac{W_{R_{ij}}^{*}}{W_{R_{i}}^{*}}$$

where *n* is the number of index layer indicators under the corresponding field layer.

Similarly, the weights $W_{R}$ of standard layer indicators and the relative weights $W_{R_{i}}$ of field layer indicators are obtained. The calculation results of risk indicator weights for the HSR project are shown in Table 4.

### 5.2. Valuation of Risk Factors

After establishing the indicator system, the risk values of index-layer indicators (risk factors) must be evaluated. In this paper, expert grading was employed to determine the risk values because objective data is difficult to obtain. In order to ensure the validity of the data, the experts involved in the grading process were managers who actually participated in the planning, construction or operation of the HSR project. The range of risk factor values in the questionnaire ranged from 0.1–0.9, where 0.1 indicates a negligible impact and 0.9 indicates a very serious impact on the project. The classification of risk values is shown in Table 5.

Therefore, the risk factor value *x* is:

(5) $$x=\frac{1}{k}\overset{k}{\underset{i=1}{\sum }}x_{i}$$

where *k* is the number of experts.

### 5.3. Calculation of Coupling Coefficient

A coupling degree model is used to measure the degree of coupling between risk factors, which can reflect the degree of interaction between elements within the system [67]. The coupling degree model included the following two steps:

Step1. Constructing an effect function. In this step, the risk values evaluated earlier are standardized and converted into efficacy coefficients. Since the risk indicators have positive effects, the effect coefficient $U_{ij}$ of each risk indicator can be expressed as:

(6) $$U_{ij}=(X_{ij}-B_{ij})/A_{ij}-B_{ij})$$

where $U_{ij}$ reflects the degree to which each indicator reaches the target, ranging within [0,1], and *i* is the order parameter of a system. In this paper, this refers to the number of a risk field in a risk subsystem (such as V_1_ within V). $X_{ij}$ is the risk value of the *j*^th^ indicator of the order parameter *i* (such as V_11_ in V_1_). $A_{ij}$ and $B_{ij}$ are the upper and lower limits of the indicator, respectively, when the system reaches a stable state.

Step 2. Constructing a coupling function. Suppose there are *m* risk indicators participating in the coupling. Considering Liu’s analysis of the value of the coordination coefficient for coupling degree models and its numerical distribution [68], the coupling degree model of *m* indexes is expressed as:

(7) $$C_{m}={\left\{\frac{U_{1}\cdot U_{2}\cdot U_{3}\cdot \cdot \cdot U_{m}}{{\left[(U_{1}+U_{2}+\ldots +U_{m})/m\right]}^{m}}\right\}}^{5}$$

where $C_{m}$ is the coupling coefficient among *m* risk indicators. Its value represents the strength of the relationship between two risk factors. According to the formula, the value of the coupling coefficient satisfies 0 ≤ $C_{m}$ ≤ 1. The coupling strengths are weakest when $C_{m}$ = 0 and strongest when $C_{m}$ = 1.

### 5.4. Completing System Dynamics Equations

Based on the process above, the weights of the indicators, values of the risk factors, and coupling coefficients are obtained. Then, combined with the risk variable types and interaction relationships in the flow-stock graph, complete SD function equations are established based on SD theory [69], as follows.

(8) $${RR}_{ij}=\sum^{}({LR}_{ij}\times C_{R_{ij}-X})\;{LR}_{ij}=INTEG({RR}_{ij},x_{R_{ij}})\;LR_{i}=\sum^{}({LR}_{ij}\times W_{R_{ij}}^{*})\;LR=\sum^{}({LR}_{i}\times {WR}_{i})\;L=\sum^{}(LR\times W_{R})$$

where R∈{V, T}, ${LR}_{ij}$ Risk level of index-layer indicator, ${RR}_{ij}$ Rate of change of the risk level of the index-layer indicator, $C_{R_{ij}-X}$ Coupling coefficient related to index-layer indicator, $x_{R_{ij}}$ Risk value of index-layer indicator, ${LR}_{i}$ Risk level of field-layer indicator, *LR* Risk level of standard-layer indicator, *L* Total risk level of project in target layer.

After inputting the SD equations into the software, the risk assessment results can be obtained through simulation and analysis. In the next section, we take the actual case of the ZW HSR project as an example to illustrate more intuitively how this model is implemented for risk assessment in HSR projects.

## 6. Model Application to the Zhengzhou-Wanzhou High-Speed Rail Project

### 6.1. Project Overview

We now use the Zhengzhou-Wanzhou (ZW) HSR project to demonstrate the model’s application. The total length of the ZW HSR is 818 km and it has an estimated investment of 104 billion yuan from the local governments of Henan, Hubei and Chongqing Provinces. The project started on 11 December 2016 and is planned to be completed and fully operational in 2022.

In the planning stage of the ZW HSR project, there was great controversy about setting up stations and selecting lines. Local governments were eager to have HSR transit to promote the rapid development of the local economy. Due to the competition for stations, social conflicts broke out in the Xinye and Dengzhou cities of Henan Province, and fierce competition occurred between the Wuxi and Wushan cities of Chongqing Province. As the ratio of bridges and tunnels is high (91.9%), the project faces great risks in its construction process. During tunnel excavation in the Henan section, geological disasters and fault phenomena such as water gushing and mud bursting occurred frequently because of the complex geological structure, taking large machinery out of operation. Furthermore, the Small Three Gorges Tunnel in the Chongqing section is the longest HSR tunnel in Asia and comprises one tube and two lanes, leading to difficult and high-risk construction. In addition, the permanent land use of the project is over 114.92 ha, and the total demolition area is 14.58 ha [70]. The heavy task of demolition is prone to cause social conflicts. The project also faces problems of solid waste treatment, as the line passes through nine nature reserves, five scenic spots, nine forest parks and six centralized drinking water reservoirs. Moreover, Daba Mountain, located in the Chongqing section of the ZW HSR project, is a key forest region and habitat, with lots of native vegetation and a variety of wildlife species under state protection.

In general, ZW HSR is a representative HSR project that faces various risks both internally and externally. It is of great significance in evaluating the project’s risks, especially the effects of coupled risks.

### 6.2. Data Collection and Processing

In order to assess the real situation of the ZW HSR project, we visited its owner (Yuwan Railway Co., Ltd.), construction unit (The Fourth Company of China Railway No. 17 Bureau Group), and the 8th Bid Project Department of Chongqing Section. Questionnaires were distributed to the senior managers of these units. The questionnaire was divided into three parts that: (1) introduced the research aims, (2) introduced the meaning of each risk factor and the scoring rules, and (3) guided the experts in scoring 24 risk factors according to the actual situation of the project and their experience. Twenty questionnaires were distributed and 15 were recovered, of which 12 were valid. The risk values of the ZW HSR project were calculated from the questionnaire data using Equation (5) and are shown in Table 6.

Then, Equations (6) and (7) were used to calculate the coupling coefficients between pairs of risk factors in the ZW HSR project. The results are shown in Table 7.

### 6.3. Simulation and Results Analysis

In the simulation, taking the invariance of the coupling coefficients into account, the total time was set to three years with a starting point of May 2020 and a time step of three months.

#### 6.3.1. Analysis of Risk Level Results

Before the assessment, it is necessary to establish a risk standard; that is, to clarify the risk rating of the HSR project and the corresponding risk level range and managing principles. We set all $x_{R_{ij}}$ (the risk value of risk factor) as the lowest value (0.1) and inputted these and their corresponding coupling coefficients derived from Equation (5) into Vensim PLE software. Through simulation, the lower limit of the total risk level of the ZW HSR at the end of the next third year is determined to be 3.75. Similarly, we set all $x_{R_{ij}}$ as the highest risk factor value (0.9) and 32.76 is obtained as the upper limit of the total risk level of the ZW HSR at the end of the next third year. Then, the total risk of the ZW HSR project is divided into five risk ratings according to the risk score classifications of the Benjamin-Graham assessment method (also known as the LEC: L (likelihood) × E (exposure) × C (criticality) method) [71]. The risk ratings are defined in Table 8.

**Figure 7:**
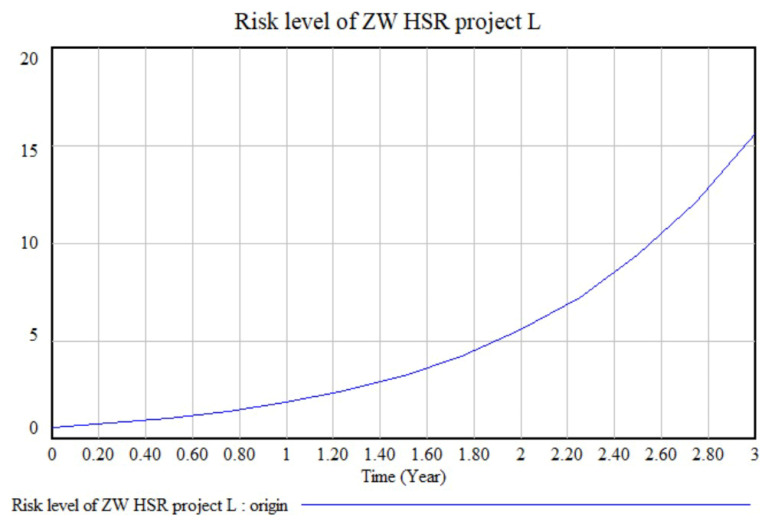
The total risk level of the Zhengzhou-Wanzhou (ZW) high-speed rail (HSR) project.

Next, the risk values determined by experts and the coupling coefficients from Section 6.2 were input into the model. Figure 7 shows a model of the future total risk level of the ZW HSR project; over time, the total risk level and its growth rate continuously increase, presenting an irreversible trend. This means that, if risks are not controlled in a timely manner, the total risk will continue to expand and the project will miss its objectives, leading to serious consequences.

To analyze the simulation results in more detail, we analysed the risk status of the ZW HSR project at the end of the next third year. At this time, the total risk level is 15.63, which means the project has a risk rating of III, with obvious risks that need to be mitigated. Furthermore, the total risk is composed of 72% vulnerability risk and 28% threatening risk, which is derived from Equation (9).

The proportion of vulnerability risk or threatening risk in the total risk is defined as $\varepsilon_{R}$, where R∈{V, T}.

(9) $$\varepsilon_{R}=(LR\times W_{R})/\sum^{\text{​}}(LR\times W_{R})$$

**Figure 8:**
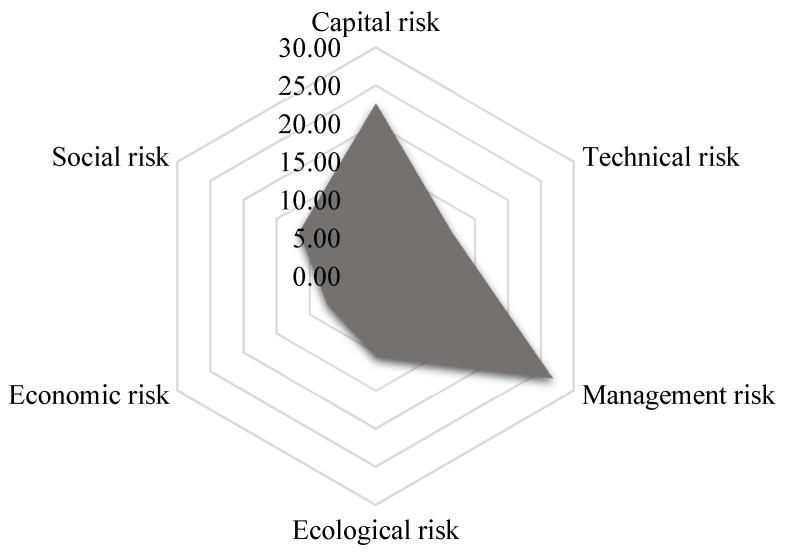
Field risk levels of the Zhengzhou-Wanzhou (ZW) high-speed rail (HSR) project.

**Figure 9:**
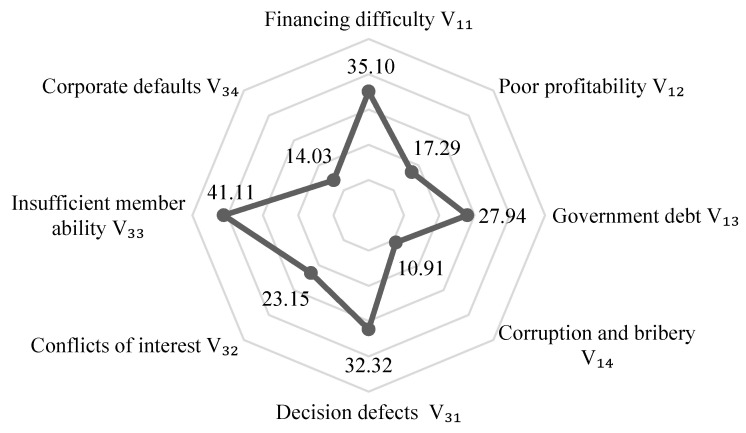
Risk levels of management and capital risk factors.

Combined with the risk level of each risk field shown in Figure 8, we can find that at this time, the threatening risk of the project, that is, the impact on the external environment (social, economic and ecological) is relatively small, although the project still poses certain threats to social stability and the ecological environment. What needs more attention is that there are great vulnerability risks, especially in terms of management problems and capital pressure. Further analyzing the levels of management and capital risk factors, Figure 9 shows that the problems of insufficient capacity of organizational members and defects in decision-making systems are prominent, and the project faces difficulties dealing with financing and government debt. In this regard, project managers should respond. On the one hand, they should focus on improving the abilities of their members and establishing a scientific and standardized decision-making system. On the other hand, the project owner department should broaden the financing channels to reduce government debt and use financial instruments such as insurance and options to transfer risks.

#### 6.3.2. Analysis of Risk Coupling Effects

After observing the overall risk level, we now analyze its sources. In the context of this paper, we focus on the role of risk coupling effects; that is, what kind of risk relationships contribute greatly to the total risk level? These key risk coupling effects are what we need to guard against. Here, the difference method is used for analysis. Suppose we aim to study the coupling effect of certain types of risk. We set the relevant coupling coefficients to 0 and hold the other parameters unchanged, forming an experimental group. The model with all complete parameters forms the control group. After two sets of models are simulated using the same total durations and time steps, the difference in risk level can reflect the contribution of this type of risk coupling to the total risk level, that is, the risk coupling effect. The greater the difference, the stronger the effect of the coupled risk. Next, we study the risk coupling effect at the end of the next third year. According to Figure 2, the coupled risks of HSR projects are divided into homogeneous and heterogeneous types.

**Figure 10:**
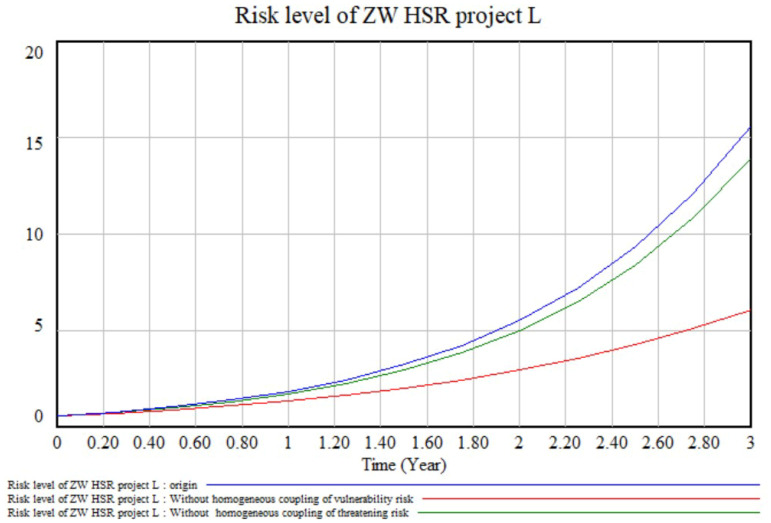
Models of homogeneous risk coupling effects.

Homogeneous risk coupling effects. Homogeneous risk coupling is the coupling within a certain risk category of vulnerability risks or threatening risks. A simulation was run after all the coupling coefficients of homogeneous vulnerability risk coupling (such as CV_11_-V_14_) were set to 0, which produced the red line in Figure 10. The ordinate difference between the blue line (control group) and red line (experimental group 1) represents the effect of homogeneous coupling of vulnerability risk factors. Similarly, the ordinate difference between the blue line (control group) and green line (experimental group 2) represents the effect of homogeneous coupling of threatening risk factors. It can be seen from Figure 10 that, at the end of the next third year, the effect of homogeneous coupling of vulnerability risk factors is obviously greater than that of threatening ones. The calculations show that if the homogeneous coupling of vulnerability risks is well controlled, the total risk of the project can be reduced by 60%. Therefore, we should pay more attention to the homogeneous coupling of vulnerability risk factors.

In the coupling of homogeneous vulnerability risks, the coupling effect of each pair of risk factors can be calculated, and the most significant effect can be obtained. Due to the many risk factors, it is difficult to compare their coupling effects by a graph. The following formula is used to quantify the effect of coupled risk factors:

(10) $$CE(A-B)=(X{|}_{t=k}-X_{CE(A-B)}{|}_{t=k})/X{|}_{t=k}$$

where CE(A−B) is the coupling effect of risk factors A and B, $X{|}_{t=k}$ is the total risk level at the end of the *k*^th^ year, and $X_{CE(A-B)}{|}_{t=k}$ represents the total risk level at the end of the *k*^th^ year after removing the coupling effect of risk factors A and B.

**Figure 11:**
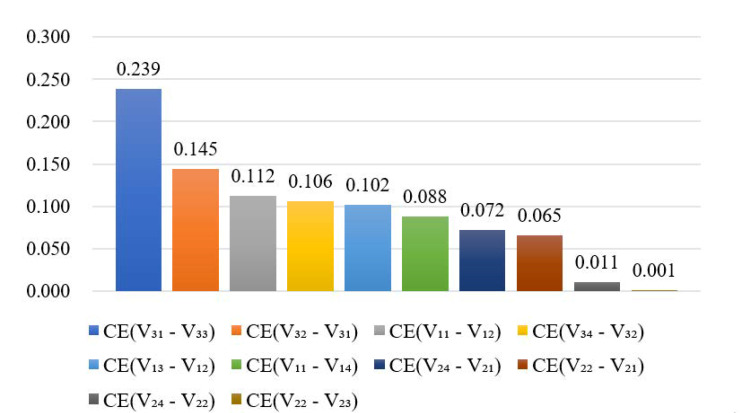
Coupling effects of homogeneous vulnerability risk pairs.

After simulation and substituting the simulation results into Equation (10), at the end of the next third year a ranking of the coupling effects of homogeneous vulnerability risks was obtained (Figure 11). According to Figure 11, the risk factor pair “V_31_-V_33_ (Decision defect - Insufficient member ability)” has a significantly stronger coupling effect than the other pairs. Summing up the conclusion in Section 6.3.1, these two risk factors have high risk levels and a strong coupling effect, so it is necessary to take steps to mitigate the risks and prevent their transmission. On one hand, the standardized and scientific decision-making procedures need to be clarified, and a reasonable organizational structure needs to be established. On the other hand, the decision-making related to the major issues of project should be led by qualified experts in consultation with third-party agencies.

**Figure 12:**
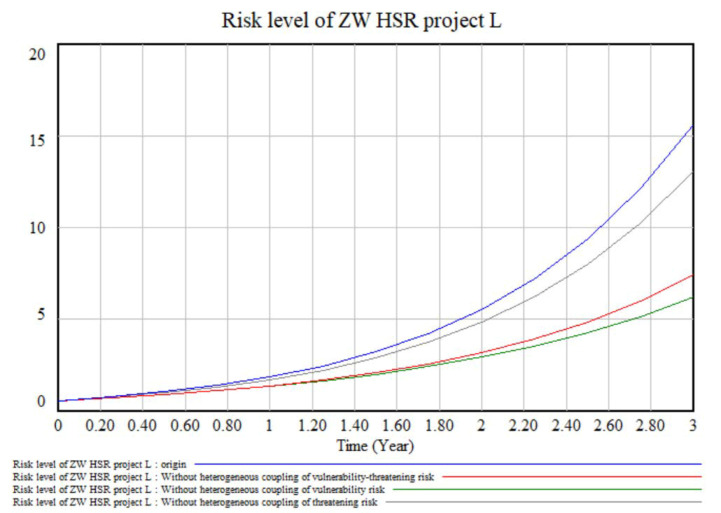
Models of heterogeneous risk coupling effects.

Heterogeneous risk coupling effects. There are three types of heterogeneous risk coupling: heterogeneous vulnerability risk coupling, heterogeneous threatening risk coupling and heterogeneous vulnerability-threatening risk coupling. In Figure 12, the grey line represents the heterogeneous coupling of threatening risk (experimental group 3), the green line represents the heterogeneous coupling of vulnerability risk (experimental group 4), and the red line represents the heterogeneous coupling of vulnerability and threatening risk (experimental group 5). Comparing the differences in risk levels between these and the control group (blue, origin), it is clear that, compared to the heterogeneous coupling of threatening risk, the heterogeneous coupling of vulnerability risk and the heterogeneous coupling of vulnerability-threatening risk need to be studied further. The key risk pairs in these two types of heterogeneous coupling are discussed next.

**Figure 13:**
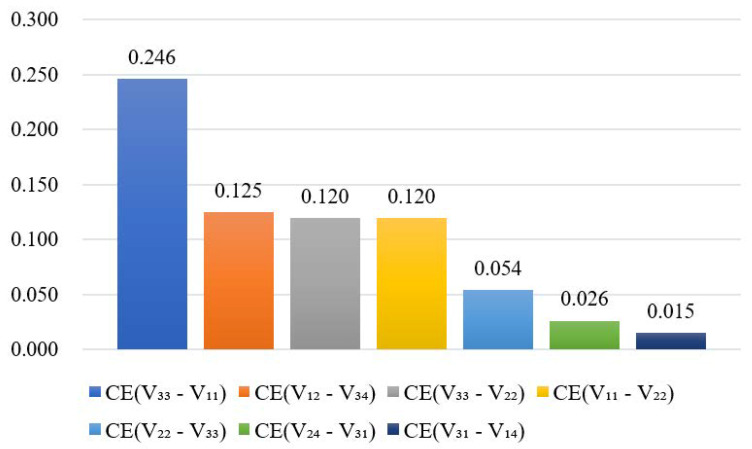
Coupling effects of heterogeneous vulnerability risk pairs.

**Figure 14:**
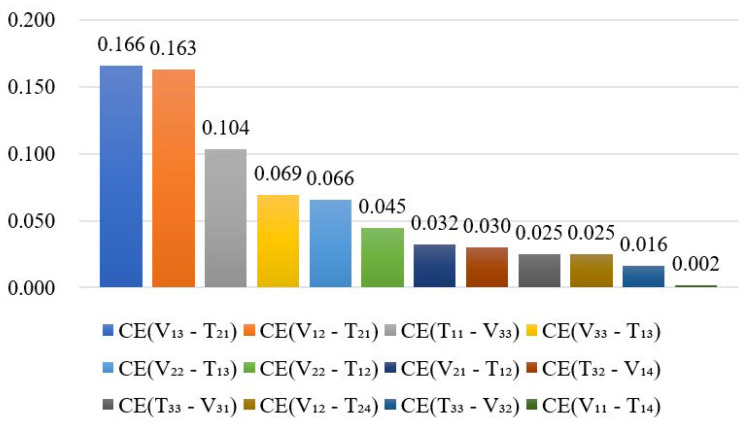
Coupling effects of heterogeneous vulnerability-threatening risk pairs.

After running simulations and substituting their results into Equation (10), rankings of the coupling effects of the heterogeneous vulnerability risk and vulnerability-threatening risk were obtained (Figure 13 and Figure 14, respectively).

Comparing Figure 13 and Figure 14, in terms of the number of coupled pairs, heterogeneous vulnerability risk has fewer pairs than heterogeneous vulnerability-threatening risk. However, the average effect of coupled vulnerability pairs is greater which, to some extent, explains why the heterogeneous vulnerability coupling effects are stronger than heterogeneous vulnerability-threatening ones.

In detail, Figure 13 shows that, of the heterogeneous vulnerability coupled pairs, the effect of “V_33_-V_11_ (Financing difficulties—Insufficient members capabilities)” is significantly stronger than the others. In this regard, an open and transparent bidding procedure and supervision system should be established to prevent the following situation: in cases of insufficient funds, departments may select contractors who are low-cost but have insufficient qualifications and abilities so they can win a tender bid. In Figure 14, according to the Pareto principle (also known as the 80–20 rule), the “V_13_-T_21_ (Unfavorable economic situation-Government debt)” and “V_12_-T_21_ (Unfavorable economic situation-Poor profitability)” pairs are the most notable vulnerability-threatening risk couples. This infers that the project is sensitive to economic fluctuations of the market. We have two suggestions: (1) comprehensive assessments of risk and profit should be made in the early stage of a feasibility study; and (2) the fare policy should be flexible according to market demand.

## 7. Conclusions and Future

This study established a model based on the SD method to assess the risks of HSR projects. This risk assessment model goes beyond existing models in that it considers the coupling effects of risk factors and reflects the internal mechanism of risk accumulation. The model can be used as a decision-making tool by HSR industry practitioners and government departments. It can comprehensively analyze the risks to HSR projects and identify their key coupling effects, thereby increasing the likelihood of success of HSR projects in complex environments. Moreover, the methods and results of this paper are transferable to the risk assessment of other major infrastructure projects. The main research findings are as follows:

- The risks of HSR projects were comprehensively identified and a multi-layer risk list was determined. Twenty-four risk factors were identified and classified into vulnerability and threatening risk subsystems and six risk categories.
- Coupled risk relationships, which are essentially causal relationships between risk factors, were analysed qualitatively. The results show that there are homogeneous and heterogeneous risk couplings within and between the vulnerability and threatening risk subsystems. This means that risk transmission within and between systems may increase the risk of a certain category or change the risk categories. A mathematical model based on SD was established that quantitatively expresses the risk-increasing effects of the risk coupling relationships on the project’s risk level (risk coupling effects).
- The model was then applied to the ZW HSR project. The results show that, at the end of the next third year: (1) the ZW HSR project has a level III risk rating with high vulnerability risk, of which management risk and capital risk are relatively high; (2) homogeneous vulnerability risk coupling, heterogeneous vulnerability risk coupling and heterogeneous vulnerability-threatening risk coupling contribute most to the total risk of the ZW HSR project, of which V_31_-V_33_, V_33_-V_11_, V_13_-T_21_, and V_12_-T_21_ are the key risk pairs. Brief risk mitigation strategies were proposed for these.

This study has certain limitations that can be optimized in further studies. First, there was unavoidable subjectivity in the experts’ risk evaluations. In the future, more quantitative indicators could be introduced as risk assessment factors. Secondly, due to the limitation of the SD method, the risk coupling analysis made in this paper mainly focused on the causality between pairs of risk factors. In future, other methods, such as N-K models, could be adopted to explore the coupling effects of multiple risk factors. Third, since the model’s risks and coupling coefficients are fixed, it is very challenging to improve the model sufficiently to make flexible long-term predictions. Last but not least, in the modeling process, most of the risks and coupling relationships were based on evidence from China and, thus, should be adapted for use in other contexts.

## Figures and Tables

**Table 1:** High-speed rail (HSR) project risk subsystems, categories and factors, with the coding system used in subsequent analysis.

Risk Subsystems	Risk Categories	Risk Factors
Vulnerability	Capital risk $V_{1}$	Financing difficulty $V_{11}$ Poor profitability $V_{12}$ Government debt $V_{13}$ Corruption and bribery $V_{14}$
	Technical risk $V_{2}$	Survey and design defects $V_{21}$ Technical complexity $V_{22}$ Different technical standard $V_{23}$ Inadequate safety protection $V_{24}$
	Management risk $V_{3}$	Decision defect $V_{31}$ Conflicts of interest $V_{32}$ Insufficient member ability $V_{33}$ Corporate defaults $V_{34}$
Threatening	Ecological risk $T_{1}$	Regional ecosystem vulnerability $T_{11}$ Complex hydrological, geological, and meteorological conditions $T_{12}$ Natural disasters $T_{13}$ Nature reserves and historic reservations $T_{14}$
	Economic risk $T_{2}$	Unfavorable economic situation $T_{21}$ Unbalanced development of regional economy $T_{22}$; Alternative transportation facilities $T_{23}$ Lower-level regional economy development $T_{24}$
	Social risk $T_{3}$	War and riot $T_{31}$ Difficulties in land acquisition, demolition and resettlement $T_{32}$
		Strong public interest appeals $T_{33}$ Public sensitivity to pollution $T_{34}$

**Table 2:** General risk assessment methods in construction projects.

Reference	Method	Features and Advantages	Limitations
[14,54]	Analytic hierarchy process	The comprehensive importance of each level factor can be obtained, making it suitable for decision-making in complex systems with multiple objectives, levels and factors.	The data is subjective and not always convincing enough, and new decision-making options are not available.
[55]	Fuzzy comprehensive evaluation	Based on the theory of fuzzy mathematics, multi-layer operations are run to determine the risk level of the project. It is suitable for the evaluation of fuzzy or qualitative risks.	The determination of factor weights is subjective and not convincing enough.
[56]	Delphi	Semi-structured questionnaires on risk are sent to a group of experts for multiple rounds until a reliable consensus is reached. Used for problems that are difficult to handle by other methods.	The results depend largely on the experts’ qualifications and number.
[57]	Monte Carlo simulation	A risk structure is established and the mathematical relationships between risk variables are expressed by a Bayesian formula. Applicable to problems with obvious causality between risks.	Large amounts of high-quality data are required to obtain a convincing risk structure and conditional risk probability distributions.
[58]	Bayesian network	The risk structure is established and the mathematical relationship between risk variables is expressed by Bayesian formula. It is applicable to the problems with obvious causality between risks.	Large amounts and high-quality data are required to obtain a convincing risk structure and conditional probability distributions of risks.
[17]	Interpretative structural modeling	An adjacency matrix is used to study the interactions between risks and establish a risk structure. Risks can be divided into several categories according to the type and intensity of their interactions.	Only suitable for qualitative risk analysis. Quantitative evaluations cannot be obtained.
[59,60]	System dynamics	A complex system with multiple variables and nonlinear characteristics can be constructed and predicted dynamically, and the interactions of the system’s components can be represented quantitatively.	It is difficult to construct a suitable dynamic model of a complex system.
[61]	N-K model	The current status of a complex system and the effects of the interactions between different components can be analyzed quantitatively.	A large amount of complete historical data is required, and the future situation cannot be predicted.

**Table 3:** Risk coupling between the vulnerability and threatening risk subsystems.

Case Description	Causal Relationship of Risk Factors	Direction of Risk Coupling
Beijing-Shanghai high-speed rail (HSR) passes through the Ming Mausoleum (a key national heritage conservation unit) and crosses Yangcheng Lake Ecological Area (an ecological protection zone). In order to defend the integrity of the sites and reduce water pollution, the original design and construction scheme of the project has been modified. The new scheme increased the length of the bridge by 5931 m and the main engineering cost increased by 29.02 million yuan. The major changes to the project have caused great financial stress for its investors.	Nature reserves and historic reservation → Financing difficulty	Ecological risk → Capital risk
During the construction of the Beijing-Shanghai HSR, costs increased significantly due to high inflation. Owing to the large amount of project loans, long construction period and rising interest rate, the debt burdens of the Ministry of Railways and of local governments increased. For example, 80.8% of the lines have used viaduct technology, for which the cost per km is 20–30 million yuan higher than that of ordinary subgrade. Half of this investment is financed by bank loans. Even if the minimum interest rate of syndicated loans at that time was 5.5%, the annual interest expense would be 6.05 billion yuan.	Unfavorable economic situation → Government debt	Economic risk → Capital risk
Poor geological engineering conditions along the Beijing-Shanghai HSR, mainly manifested as soft soil, make its construction difficult.	Complex hydrological, geological, and meteorological conditions → Technical complexity	Ecological risk → Technical risk
During the construction of the Beijing-Shanghai HSR, the design institutes did not complete the design work in time according to the environmental impact assessment (EIA) document, which affected the implementation of the environmental protection measures. Additionally, the construction enterprises did not carry out unified treatment of waste soil according to their contracts, instead using it to fill ponds and mountain gullies, thus causing air and water pollution, which is harmful to the local water and soil environments.	Corporate defaults → Regional ecosystem vulnerability	Management risk → Ecological risk
Due to a lack of public participation in the HSR planning system during the planning of the Shanghai-Wuhan-Chengdu HSR and Shanghai-Kunming HSR, members of the public spontaneously gathered for demonstrations and caused mass incidents to express their strong appeal for the implementation of HSR transit in their areas.	Decision defect → Strong public interest appeals	Management risk → Social risk
During the planning of the Dazhou-Chongqing HSR, there was a dispute between the East Line and West Line, which caused local governments to compete for the project. Among them, the governments of Dazhou and Guang’an made different statements regarding the line chosen for HSR on their information websites, which aroused dissatisfaction and strong appeals from the public.	Conflicts of interest → Strong public interest appeals	Management risk → Social risk
Due to the continental plate on which Indonesia is located, earthquakes and volcanic eruptions are frequent. Indonesia’s Ministry of Transport have stressed twice that, if earthquake resistance requirements cannot be met, construction of the Jakarta-Bandung HSR project will not be permitted. Such natural disasters pose challenges to existing design and construction technology.	Natural disasters → Technical complexity	Ecological risk → Technical risk
Compensation for land acquired for the Zhengzhou-Xuzhou HSR was embezzled by local officials. Because the compensation did not meet state standards, this hurt the interests of the people and resulted in petitions.	Corruption and bribery → Difficulties in land acquisition, demolition and resettlement	Capital risk → Social risk
As local people may lack the capacity and willingness to pay for tickets, only about 30 pairs of HSR Electric Multiple Units (EMUs) of Zhengzhou-Xi’an HSR operates every day, with an attendance rate of less than 50%. After two years’ operation, in 2016, this HSR line lost 1.4 billion yuan, leaving the Zhengzhou Railway Bureau at a loss of 1 billion yuan.	Lower-level regional economy development → Government debt	Economic risk → Capital risk
A serious epidemic influenced the macro-economies of various countries. Basic economic activity nearly collapsed and market demand for HSR was almost non-existent. The volume of passenger traffic in the Beijing-Shenyang HSR was less than 10%, and ticket revenue could not support the high operating cost.	Unfavorable economic situation → Poor profitability	Economic risk → Capital risk
The Fujian section of the Ganzhou-Longyan HSR is located in the mountains of Northwest Fujian. Carbonate rock and karst are widely distributed, and the erosional surface is dissected deeply by valleys. It is difficult to carry out accurate surveys, resulting in the hidden danger of engineering design defects.	Complex hydrological, geological, and meteorological conditions → Survey and design defects	Ecological risk → Technical risk
China Railway Corporation’s financial report shows that, up to September 2018, the company’s debt was 5.28 trillion yuan, which is mainly borne by the Railway Bureau and local governments. Experts have stated that once the debt risk is out of control, it may cause systemic financial risk.	Government debt → Unfavorable economic situation	Capital risk → Economic risk
The California HSR project is located on the Pacific Plate, which has high seismic activity. Generally speaking, HSR needs rigid structures to meet strict limits of deflection and vibration control. However, in earthquake areas, flexible and ductile structures are more earthquake-proof, which brings additional challenges to the designers and constructors of this project.	Natural disasters → Insufficient member ability	Ecological risk → Management risk

**Table 4:** Weights of risk indicators.

Target Layer Indicator (*L*)	Standard Layer Indicators ( R)	Field Layer Indicators ( $R_{i}$)	Index Layer Indicators ( $R_{ij}$)	Mean Value (MS)	Absolute Weights of $R_{ij}$ ($W_{R_{ij}}^{*}$)	Relative Weights of $R_{ij}$ ($W_{R_{ij}}$)
Risk of HSR project	Vulnerability risk $W_{V}$ = 0.553	Capitalrisk risk V_1_ $W_{V_{1}}$ = 0.374	Financing difficulty $V_{11}$	4.125	0.055	0.266
			Poor profitability $V_{12}$	3.267	0.044	0.212
			Government debt $V_{13}$	3.625	0.049	0.237
			Corruption and bribery $V_{14}$	4.400	0.059	0.285
		Technical risk $W_{V_{2}}$ = 0.325	Survey and design defects $V_{21}$	4.400	0.059	0.328
			Technical complexity $V_{22}$	3.500	0.047	0.261
			Different technical standard $V_{23}$	3.267	0.044	0.244
			Inadequate safety protection $V_{24}$	2.215	0.030	0.167
		Management risk $W_{V_{3}}$ = 0.301	Decision defect $V_{31}$	3.625	0.049	0.295
			Conflicts of interest $V_{32}$	3.375	0.045	0.271
			Insufficient member ability $V_{33}$	2.250	0.030	0.181
			Corporate defaults $V_{34}$	3.125	0.042	0.253
	Threatening risk $W_{T}$ = 0.447	Ecological risk $W_{T_{1}}$ = 0.326	Regional ecosystem vulnerability $T_{11}$	3.533	0.047	0.324
			Complex hydrological, geological, and meteorological conditions $T_{12}$	3.067	0.041	0.283
			Natural disasters $T_{13}$	2.375	0.032	0.221
			Nature reserves and historic reservations $T_{14}$	1.875	0.025	0.172
		Economic risk $W_{T_{2}}$ = 0.342	Unfavorable economic situation $T_{21}$	3.667	0.049	0.32
			Unbalanced development of regional economy $T_{22}$	2.375	0.032	0.209
			Alternative transportation facilities $T_{23}$	2.215	0.030	0.196
			Lower-level regional economy development $T_{24}$	3.133	0.042	0.275
		Social risk $W_{T_{3}}$ = 0.332	War and riot $T_{31}$	2.533	0.034	0.228
			Difficulties in land acquisition, demolition and resettlement $T_{32}$	4.000	0.054	0.362
			Strong public interest appeals $T_{33}$	1.750	0.024	0.161
			Public sensitivity to pollution $T_{34}$	2.750	0.037	0.249

**Table 5:** Risk value classification.

Risk Value	0.1	0.3	0.5	0.7	0.9
Degree of impact	Negligible	Low	Medium	Serious	Very serious

**Table 6:** Values of risk factors in the Zhengzhou-Wanzhou (ZW) high-speed rail (HSR) project.

Risk Factor	Risk Value	Risk Factor	Risk Value
Financing difficulty $V_{11}$	0.77	Regional ecosystem vulnerability $T_{11}$	0.64
Poor profitability $V_{12}$	0.75	Complex hydrological, geological, and meteorological conditions $T_{12}$	0.76
Government debt $V_{13}$	0.67	Natural disasters $T_{13}$	0.54
Corruption and bribery $V_{14}$	0.67	Nature reserves and historic reservations $T_{14}$	0.23
Survey and design defects $V_{21}$	0.60	Unfavorable economic situation $T_{21}$	0.45
Technical complexity $V_{22}$	0.90	Unbalanced development of regional economy $T_{22}$	0.31
Different technical standard $V_{23}$	0.19	Alternative transportation facilities $T_{23}$	0.29
Inadequate safety protection $V_{24}$	0.42	Lower-level regional economy development $T_{24}$	0.41
Decision defect $V_{31}$	0.79	War and riot $T_{31}$	0.26
Conflicts of interest $V_{32}$	0.84	Difficulties in land acquisition, demolition and resettlement $T_{32}$	0.51
Insufficient member ability $V_{33}$	0.60	Strong public interest appeals $T_{33}$	0.48
Corporate defaults $V_{34}$	0.85	Public sensitivity to pollution $T_{34}$	0.35

**Table 7:** Coupling coefficients of Zhengzhou-Wanzhou (ZW) high-speed rail (HSR) project risk factors.

Coupled Risk Factors	Coupling Coefficient	Coupled Risk Factors	Coupling Coefficient
CV_11_-V_14_	0.976	CV_31_-V_33_	0.910
CV_11_-T_14_	0.178	CV_31_-V_14_	0.166
CV_11_-V_22_	0.966	CV_32_-V_31_	0.995
CV_11_-V_12_	0.999	CV_33_-V_11_	0.925
CV_12_-T_24_	0.428	CV_33_-V_22_	0.806
CV_12_-T_21_	0.940	CV_33_-T_13_	0.986
CV_13_-V_12_	0.984	CV_34_-V_32_	0.839
CV_13_-T_21_	0.821	CT_11_-V_33_	0.794
CV_14_-V_34_	0.932	CT_11_-T_13_	0.965
CV_21_-T_12_	0.933	CT_13_-T_12_	0.865
CV_22_-V_23_	0.061	CT_21_-V_13_	0.821
CV_22_-V_21_	0.806	CT_21_-T_13_	0.959
CV_22_-T_12_	0.960	CT_23_-T_24_	0.861
CV_22_-T_13_	0.714	CT_32_-T_31_	0.573
CV_24_-V_21_	0.854	CT_32_-V_14_	0.911
CV_24_-V_22_	0.482	CT_33_-V_31_	0.736
CV_24_-V_31_	0.612	CT_34_-T_11_	0.639
CV_24_-V_33_	0.854	CT_21_-T_31_	0.690
CT_33_-V_32_	0.679	-	-

**Table 8:** The risk standard of Zhengzhou-Wanzhou (ZW) high-speed rail (HSR) project.

Risk Rating	Interval of Risk Level	Specified Interval Value of Risk Level	Risk Management Principle
V	(0, 20%)	(3.75, 9.55)	There are acceptable minor risks and the project can be carried out normally
IV	(20%, 40%)	(9.55, 15.36)	There are general risks that should be attended to
III	(40%, 60%)	(15.36, 21.16)	There are obvious risks and mitigation measures should be taken
II	(60%, 80%)	(21.16, 26.96)	The project is highly risky and needs to be modified immediately
I	(80%, 100%)	(26.96, 32.7)	The project is extremely risky and needs to be suspended, with risk control measures implemented immediately
